# Evaluation of the efficacy of transcranial direct current stimulation in the treatment of cognitive symptomatology in the early stages of psychosis: study protocol for a double-blind randomized controlled trial

**DOI:** 10.1186/s13063-019-3288-5

**Published:** 2019-04-05

**Authors:** Thais Rabanea-Souza, Sheila M. C. Cirigola, Cristiano Noto, July S. Gomes, Caroline C. Azevedo, Ary Gadelha, Quirino Cordeiro, Álvaro M. Dias, Acioly L. T. Lacerda

**Affiliations:** 1 LiNC - Laboratório Interdisciplinar de Neurociências Clínicas, Department of Psychiatry, Universidade Federal de São Paulo, Sao Paulo, Brazil; 2 Center for Neuromodulation Studies, Department of Psychiatry, Universidade Federal de São Paulo, Sao Paulo, Brazil; 3 Department of Psychiatry, Faculdade de Ciências Médicas da Santa Casa de São Paulo, Sao Paulo, Brazil; 4 Center for Research and Clinical Trials Sinapse-Bairral, Instituto Bairral de Psiquiatria, Itapira, Brazil

**Keywords:** Early stages of psychosis, Schizophrenia, Neuromodulation, Transcranial direct current stimulation, Neuropsychology, Cognitive symptomatology, Double-blind randomized controlled trial

## Abstract

**Background:**

Cognitive deficits are core symptoms of schizophrenia that occur from the early stages of the disorder. There is reliable evidence that cognitive deficits are associated with outcomes in schizophrenia; thus, early treatment could be particularly important. Studies with different neuromodulation techniques involving subjects with schizophrenia suggest that application of transcranial direct current stimulation (tDCS) with inhibitory stimulation over the left temporo-parietal cortex and excitatory stimulation over the left dorsolateral prefrontal cortex could ameliorate positive, negative, and cognitive symptoms.

The aim of the present study protocol is to evaluate the efficacy of tDCS in the treatment of cognitive symptomatology in the early stages of psychosis.

**Methods/design:**

Seventy patients in the early stages of psychosis will be randomly allocated to receive 20 min of active 2-mA tDCS or sham stimulation once a day for 10 consecutive weekdays. The anode will be placed over the left dorsolateral prefrontal cortex and the cathode over the left temporo-parietal cortex. Neuropsychological and psychiatric assessments will be performed at baseline and at 1 and 3 months following the end of the intervention (sustained effect).

**Discussion:**

The development and utilization of potentially effective neuroenhancement tools such as the non-invasive brain stimulation technique tDCS for the treatment and rehabilitation of cognitive impairment in the early stages of schizophrenia may contribute to improving outcomes of the disorder and eventually provide a further understanding of the nature of the complex and dynamic neural processes underlying those abnormalities.

**Trial registration:**

ClinicalTrials.gov, NCT03071484. Registered on 7 March 2017.

**Electronic supplementary material:**

The online version of this article (10.1186/s13063-019-3288-5) contains supplementary material, which is available to authorized users.

## Background

### Scientific background and explanation of rationale

Schizophrenia, the most common condition among psychotic disorders, is a serious mental disorder that has a great impact on public health, accounting for about 10% of disability among persons aged 15–44 [28] and reduced life expectancy by more than 15 years [3]. The lifetime prevalence of schizophrenia is estimated at between 0.5 and 1% in the general population, with a slightly higher rate for males [4].

Different sources of evidence support the hypothesis that the etiology of psychoses is multifactorial and includes genetic, environmental, cognitive, and psychosocial factors [8]. Usually, the clinical manifestations occur between late adolescence and early adulthood. In recent decades, these manifestations have been considered premorbid stages in the description of the clinical history of psychosis, as different studies have suggested that early interventions can positively impact the prognosis of the disease [4].

Over the past 20 years there has been a major shift in the paradigm of how we understand psychoses. Consistent evidence addresses these diseases with complex genetic and multifactorial etiology as being associated with brain changes that occur throughout neurodevelopment, and not just as processes that settle and progress with the onset of symptoms (neurodegeneration) [1]. Psychosis mechanisms begin to be understood as due to abnormal changes in brain circuits that culminate in clinical manifestations of the disease which, in turn, establish progress and neurodegenerative processes related to their course. From this perspective, a model of clinical stages was developed which provides for a gradual transition from normal (stage 0) to the disease state (stage 2), including a prodromal stage (stage 1) in which symptoms are non-specific, heterogeneous, and unstable in time, coalescing and progressively differentiating to culminate in the typical feature of the disease [19]. This model represents a break in the current dichotomy between normality and disease, enabling the development of effective strategies for primary, secondary, and tertiary stages. The model has motivated a growing interest in finding specific biomarkers of steps leading to the manifestation of psychosis, to implement early intervention with the potential to improve prognosis by reducing the mortality and morbidity associated with the disease.

Different studies have shown that cognitive impairments are present before the onset of schizophrenia, remain after remission of psychosis symptoms, and can often be found in first-degree unaffected relatives, suggesting that neurocognitive alterations are a feature of psychosis which influence social and interpersonal skills, leading to an impairment of social cognition. This explains much of the variance-related functional impairment [12–15].

Those findings have encouraged a proliferation of research on neurocognitive enhancement aiming at the development of potentially effective treatment and rehabilitation of cognitive symptoms in patients with schizophrenia. In this scenario, characterized by a growing interest in developing novel neuroenhancement techniques, implementing non-invasive brain stimulation techniques aroused a new age in systems neuroscience research in which research examining the modulation of cognition using transcranial direct current stimulation (tDCS) is one of the most rapidly advancing fields in cognitive neuroscience. Recent studies have demonstrated significant, often strong, effects of tDCS on cognitive processes [10].

The tDCS is a non-invasive neuromodulatory technique that delivers low-intensity, direct current to cortical areas facilitating or inhibiting spontaneous neuronal activity and its plasticity [7, 27]. Although there are many brain stimulation techniques, tDCS is a low-cost and portable method that is well tolerated by participants, especially when compared with other techniques. In addition, there is no report of serious risks associated with treatment with tDCS in the literature. Possible adverse events related to the method usually include mild and transient conditions which do not require specific intervention, such as local redness and discomfort, mild headache, and, in rare cases, minor burns at the electrode placement site. However, all these events can be easily avoided by monitoring during the procedure [10, 24].

In patients with schizophrenia, tDCS has been demonstrated to be potentially useful for the treatment of positive symptoms [6] as well as cognitive symptoms [26]. Recent findings suggest that repetitive stimulation over specific time intervals may be a promising option to increase the efficacy of tDCS [24]. Additionally, Palm et al. [26] reported an improvement of 25% of the negative symptomatology in a case report study, and these results reflected a reduction of 12% in the total score of the Positive and Negative Syndrome Scale (PANSS).

Despite the established efficacy in the treatment of psychotic (positive) symptomatology, antipsychotics have been largely ineffective in treating cognitive deficits and negative symptoms associated with psychoses [21]. Neuropsychological deficits are present since the onset of the disease [2] and are considered nuclear symptoms in psychosis, exhibiting a marked impact on overall functioning [5]. Robust evidence suggests that the longer the time to start treatment, the worse the clinical evolution of the patient, reinforcing the importance of early diagnosis and treatment [11].

The efficacy of tDCS in improving cognitive deficits in both neurological and psychiatric conditions has been amply demonstrated in the literature. As cortical stimulation has a positive effect on short-term cognition, studies emphasized that stimulation may bring benefits to the potential of rehabilitation in patients with cognitive impairment as well [23]. However, as far as we know, there is no study examining the effects of tDCS on cognition in the early stages of psychosis.

Given the importance of reentering individuals with schizophrenia to the community, the Measurement and Treatment Research to Improve Cognition in Schizophrenia (MATRICS) initiative of the US National Institute of Mental Health (NIMH) set the development of a Consensus Cognitive Battery (MCCB) for measuring cognition in schizophrenia, aiming to guide the design of clinical trials for cognition-enhancing agents and encouraging new research [16–18], which will be used in this present study protocol.

### Objectives

The aim of the present study protocol is to evaluate the efficacy of tDCS in the treatment of cognitive symptomatology in the early stages of psychosis. We hypothesized that the patients randomly allocated to the experimental group will demonstrate a significant better performance on cognitive processes in comparison to those in the matched control group.

## Methods/design

### Trial design

The design of the present study protocol is a double-blind, placebo-controlled randomized clinical trial.

After being randomly allocated, the experimental group will receive 20 min of active 2-mA tDCS once a day for 10 consecutive weekdays. The control group will receive a sham stimulation (placebo); the chosen parameters are that, after 40 s of real stimulation (2 mA), only a small current pulse occurs every 550 ms (110 mA over 15 ms) through the remainder of the 20-min period.

Neuropsychological and psychiatric assessments will be performed at the time of consent (baseline) and at 1 and 3 months following the end of the intervention (maintenance effect). The primary outcome will be cognitive function; the secondary outcomes will be the positive and negative symptoms. Outcome assessments will be performed by trial research staff. Primary and secondary outcome assessors (neuropsychologists and psychiatrists) and patients will be blinded to randomized allocation after assignment to the interventions.

**Fig. 1:**
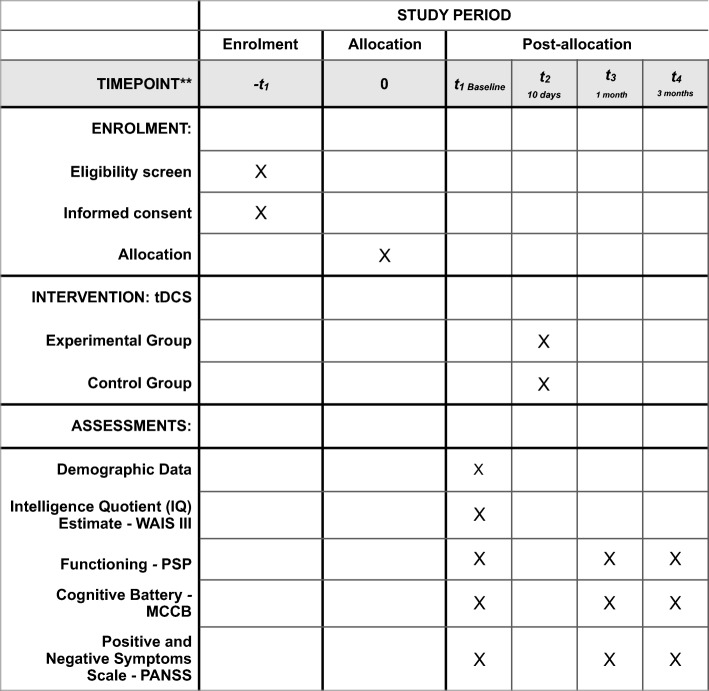
Schedule of the trial interventions and assessments

**Fig. 2:**
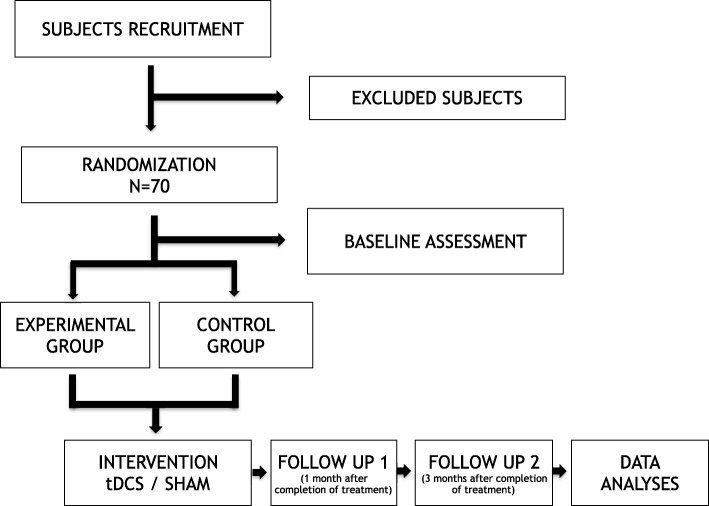
Flowchart of the trial

The present study protocol was written in compliance with the Standard Protocol Items: Recommendations for Interventional Trials (SPIRIT) 2013 [9]. A completed SPIRIT checklist is available as a supplement (Additional file 1), and the schedule of this study is presented in Fig. 1. The study flowchart is shown in Fig. 2.

### Participants

Recruitment to the trial will be at the Schizophrenia Program (PROESQ), Department of Psychiatry, Federal University of São Paulo (UNIFESP), Sao Paulo, Brazil.

The inclusion criteria include the following: (1) subjects of both genders, diagnosed with schizophrenia in early stage psychosis (first 5 years of illness), confirmed through the Structured Clinical Interview for the American Psychiatric Association Diagnostic and Statistical Manual of Mental Disorders, 5th edition (SCID-5); (2) aged 18–60 years; (3) minimum of 4 years of education; (4) intelligence quotient (IQ) from low average to higher scores (IQ > 70); and (5) the subjects should be receiving stable doses of antipsychotics for at least 4 weeks (antipsychotic dose stability criterion).

The exclusion criteria include (1) presence of a history of cranioencephalic trauma with loss of consciousness with a time greater than 5 min; (2) history of central nervous system diseases that affect the brain; (3) unstable clinical conditions; (4) use of drugs that affect cognitive performance such as benzodiazepines and anticholinergic agents; (5) current diagnosis of substance abuse; (6) history of substance dependence in the last 6 months, except nicotine addiction; (7) current diagnosis of another Axis I condition, confirmed through SCID-5.

Regular meetings with staff will be performed in order to clarify the study design and population. The research team will regularly contact the participants by phone to improve retention.

### Intervention

We have chosen tDCS, a non-invasive brain stimulation technique that involves the passage of a small current through the scalp and skull to modulate brain activity [10]. The electric current will be delivered by an electric stimulator (Striat, Ibramed Indústria Brasileira de Equipamentos Médicos Ltd., Sao Paulo, Brazil). In this study protocol, saline solution will be applied to sponge electrodes, which will be placed according to the international 10–20 electrode placement system. The anode will be placed with the middle of the electrode over a point midway between F3 and FP1 (left prefrontal cortex: dorsolateral prefrontal cortex, assumed to correspond to a region including Brodmann areas [BA] 8, 9, 10, and 46, depending on the subject), and the cathode will be placed over a point midway between T3 and P3 (left temporo-parietal junction, assumed to correspond to a region including BA 22, 39, 40, 41, and 42). In accordance with recent studies of tDCS in schizophrenia, the stimulation level will be set at 2 mA for 20 min. The stimulation sessions will be conducted once a day for 10 consecutive weekdays. The chosen sham/placebo stimulation parameters for the control group will be the following: after 40 s of real stimulation (2 mA), only a small current pulse will occur every 550 ms (110 mA over 15 ms) through the remainder of the 20-min period [6, 29].

No changes will be required in the ongoing treatments with other professionals, and the subject may request to discontinue at any time. Although there is no description of serious risks associated with treatment with transcranial direct current stimulation in the literature, possible adverse events related to the method, including mild and transient conditions, which do not require specific intervention, may occur*.* In case of any persistent adverse event, participants will be treated by staff of PROESQ, Department of Psychiatry, UNIFESP, Sao Paulo, Brazil.

To avoid any harm, subjects will be monitored by a trained staff from the Center for Neuromodulation Studies, Universidade Federal de São Paulo, during the procedure. Subjects will be instructed to inform of any discomfort, so the power can be turned off immediately.

### Outcome measures

At all assessment interviews, including neuropsychological and psychiatric, at the time of consent (baseline), and at 1 and 3 months following the end of the intervention (maintenance effect), IQ estimate and functioning will be tested in addition to the collection of standard demographic information. For this purpose, the Wechsler Adult Intelligence Scale Third Edition (WAIS-III) and the Personal and Social Performance Scale (PSP) will be used.

The WAIS-III is a standardized instrument for measuring the intellectual functioning and overall capacity of an individual [30, 31]. In order to measure the estimated IQ of the subjects under study, two WAIS-III subtests were selected, one to measure verbal and crystallized intelligence (Vocabulary) and another to measure non-verbal and fluid intelligence (Matrix Reasoning).

The Vocabulary subtest of the WAIS-III is a measure of verbal knowledge and crystallized intelligence. The version that will be used consists of 33 items. The task is to define a certain term, for example, “what is a BED?”. The examinee must include in his answer essential characteristics that define the specified term. Simple correct answers, usually involving a concrete aspect of the term, are scored as score 1, and correct complex answers receive score 2. Incorrect answers are scored as 0 [31].

The Matrix Reasoning subtest of the WAIS-III is a measure of abstract reasoning and execution and fluid intelligence. The subtest has 26 items, plus the three that serve as examples. It consists of four types of items: continuous and discrete patterns, classification, analog reasoning, and serial reasoning. The task requested of the examinee is to complete a series of incomplete patterns, pointing out or saying the correct answer number among the five alternatives presented [31].

The PSP is a validated clinician-administered scale that measures personal and social functioning in the domains of (1) socially useful activities, including work and study; (2) personal and social relationships; (3) self-care; and (4) disturbing and aggressive behaviors [22].

#### Primary outcome

The primary outcome will be cognitive functioning, which will be assessed by the MCCB for measuring cognition in schizophrenia. The MCCB embraces the main cognitive domains affected in schizophrenia [25], namely (1) speed of processing measured by *Brief Assessment of Cognition in Schizophrenia* (BACS): *Symbol Coding* (a timed paper-and-pencil test in which the respondent uses a key to write digits that correspond to nonsense symbols), *Category Fluency: Animal Naming* (a test in which the respondent names as many animals as she/he can in 1 min), and the *Trail Making Test*: Part A (a timed paper-and-pencil test in which the respondent draws a line to connect consecutively numbered circles placed irregularly on a sheet of paper); (2) attention/vigilance measured by the *Continuous Performance Test-Identical Pairs* (CPT-IP), a computer-administered measure of sustained attention in which the respondent presses a response button to consecutive matching numbers; (3) working memory measured by the *Wechsler Memory Scale*®*-3rd Edition* (WMS®-III): *Spatial Span* (using a board on which 10 cubes are irregularly spaced, the respondent taps cubes in the same (or reverse) sequence as the test administrator) and *Letter*–*Number Span* (orally administered test in which the respondent mentally reorders strings of numbers and letters and repeats them to the administrator); (4) verbal learning measured by the *Hopkins Verbal Learning Test-Revised*™ (HVLT-R™), which is an orally administered test in which a list of 12 words from three taxonomic categories is presented and the respondent is asked to recall as many as possible after each of three learning trials; (5) isual learning measured by the *Brief Visuospatial Memory Test-Revised* (BVMT-R™), which is a test that involves reproducing six geometric figures from memory; (6) reasoning and problem solving measured by the *Neuropsychological Assessment Battery*® (NAB®), which consists of mazes; the task is composed of seven timed paper-and-pencil mazes of increasing difficulty that measure foresight and planning; (7) *Mayer-Salovey-Caruso Emotional Intelligence Test* (MSCEIT™), a paper-and-pencil multiple-choice test that assesses how people manage their emotions.

#### Secondary outcomes

The secondary outcomes will be positive and negative symptoms. Symptom severity will be assessed by means of the Positive and Negative Syndrome Scale (PANSS) [20]. Trained psychiatrists will conduct all interviews.

### Power and sample size calculation

The sample size calculation was performed by using the *T* statistic and non-centrality parameter. The expected effect size of 0.8 was conservatively estimated on the basis of the results of more recently published studies [10]. The basis of the sample size estimation is an expected cognitive improvement of about 35% in the tDCS intervention group. The sample size was calculated assuming an aimed power of 80% and a standard deviation of the outcome in the population of 1.0 (standard normal deviate for α = Zα = 1.95996; standard normal deviate for β = Zβ = 0.84162). After consideration of a non-adherence rate of 10% and a drop-out rate of 10% in both arms, the case number results in 70 patients (35 patients per group).

### Group allocation

Subjects will be allocated to the experimental and control groups in a 1:1 ratio, by using the sequence generated by http://www.randomisation.com. The allocation sequence will be generated by a technician who will not be involved in the neuropsychological or psychiatric assessments. The staff in charge of delivering the tDCS/sham intervention will be informed of the treatment allocation.

### Blinding

Application of the assigned treatment will be carried out after the neuropsychological and psychiatric assessments. A double-blinding arrangement will be conducted in this clinical trial. Primary and secondary outcomes raters (neuropsychologists and psychiatrists) and patients will be blinded to randomized allocation after assignment to interventions. The tDCS/sham intervention will be delivered by an independent research staff.

### Ethical approval

This study protocol was approved by the Ethics Committee of the Federal University of Sao Paulo, in Sao Paulo, Brazil, and written informed consent will be obtained from all participants in the trial (see Additional file 2).

The results will be presented at scientific meetings and published in periodicals.

### Statistical analyses

The data will be analyzed with the Statistical Package for Social Sciences (SPSS 18.0, Chicago, IL, USA). Statistical significance will be set at *p* < 0.05 (two-tailed) for all analyses.

Student’s *t* test will be used in comparisons involving continuous clinical and sociodemographic variables with normal distribution and the Mann-Whitney test for comparisons involving clinical and sociodemographic data with non-normal distribution. Comparisons involving categorical variables will be performed using the chi-square test.

Analysis of Covariance (ANCOVA) with repeated measures, with intervention group used as intersubject factor, neuropsychological scores from MCCB tests as intrasubject factors, and years of education as a covariate, will be performed to evaluate the efficacy of intervention in the treatment of cognitive deficits (primary outcome). The same analysis, using PANSS negative and positive subscale scores as intrasubject factors and duration of illness as a covariate, will be performed to evaluate the efficacy of intervention in the treatment of negative and positive symptoms (primary outcome).

### Data monitoring

The trial is externally monitored (Laboratório Interdisciplinar de Neurociências Clínicas, Department of Psychiatry, Universidade Federal de São Paulo, Sao Paulo, Brazil) in accordance with Good Clinical Practice (GCP) recommendations.

### Confidentiality

Information about study subjects is kept confidential. All data are entered into a dedicated study data management system, and in all data documents study subjects are assigned an individual identifying code which does not contain identifying information.

## Discussion

Historically, psychiatric treatments have focused on attenuation of established symptoms, reductions of deficits, and health restoration, neglecting strategies related to promotion of competencies. Identification and correction of abnormalities associated with worse outcomes in early stages of disease have recently assumed an important role in an emerging preventive medicine, whose concepts have been gradually incorporated by psychiatry. In this sense, it is conceivable to hypothesize that rehabilitation of cognitive deficits in the early stages of schizophrenia might improve both clinical and functioning outcomes.

Cognitive deficits are core symptoms of schizophrenia and have been associated with marked impairment in global functioning [5]. Despite the unquestionable efficacy of antipsychotic drugs in the treatment of positive symptomatology, they have demonstrated no efficacy in treatment of other symptom dimensions, including cognitive deficits, which are present even before the beginning of disease [2, 21].

The development and utilization of potentially effective neuroenhancement tools such as non-invasive neuromodulation techniques for the treatment and rehabilitation of cognitive impairment in the early stages of schizophrenia may contribute to improve outcomes of disorder and eventually further our understanding of the nature of the complex and dynamic neural processes underlying those abnormalities. Transcranial direct current stimulation has proven to be safe and efficacious as a cognitive enhancement strategy for healthy subjects [7, 10]. Additionally, increasing evidence has suggested that tDCS is potentially helpful in the treatment of different symptom dimensions in schizophrenia, including cognitive deficits [6].

### Trial status

The Protocol Version is 2.0. The trial is registered at ClinicalTrials.gov with identifier number NCT03071484.

Secondary identifying numbers include the Universal Trial Number (UTN) U1111-1217-4425; ReBec (Registro Brasileiro de Ensaios Clínicos) - Brazilian Clinical Trials Registry RBR-2T3HFV.

This trial is currently ongoing. The recruitment of subjects is expected to finish in 2019.

## Additional files

Additional file 1: SPIRIT 2013 checklist: recommended items to address in a clinical trial protocol and related documents*. (DOCX 42 kb)

Additional file 2: Written informed consent form. (DOCX 22 kb)

## Abbreviations

IQ: Intelligence quotient (IQ)
MATRICS: Measurement and Treatment Research to Improve Cognition in Schizophrenia
MCCB: MATRICS Consensus Cognitive Battery
PANSS: Positive and Negative Syndrome Scale
PSP: Personal and Social Performance Scale
SCID-5: Structured Clinical Interview for Diagnostic and Statistical Manual of Mental Disorders, 5th edition
tDCS: Transcranial direct current stimulation
WAIS-III: Wechsler Adult Intelligence Scale Third Edition
