# Supplementary material for: Evaluation of the efficacy of transcranial direct current stimulation in the treatment of cognitive symptomatology in the early stages of psychosis: study protocol for a double-blind randomized controlled trial
Source: Trials. 2019 Apr 5;20:199. doi: 10.1186/s13063-019-3288-5 (PMC6451248; doi:10.1186/s13063-019-3288-5)
Supplement: Supplementary file 2 — Written informed consent form. (DOCX 22 kb) [file 13063_2019_3288_MOESM2_ESM.docx]

**Informed Consent Form**

You are being invited to participate in a study entitled **"Evaluation of the efficacy of Transcranial Current Stimulation in the treatment of cognitive symptomatology in the early stages of psychosis",** which aims to evaluate the effectiveness of transcranial direct current stimulation (a method of painless brain stimulation performed by placing electrodes on the individual's head) on the cognitive symptomatology in individuals in the early stages of psychosis.

Your participation in this research is voluntary and your refusal to participate or drop out during the study will not lead to any problems related to your treatment. The research will involve psychiatric evaluation (positive and negative symptoms) and neuropsychological assessment (tests measuring intelligence, attention, memory, processing speed, reasoning and problem solving) through specific scales and tests, besides performing transcranial direct current stimulation procedure, previously described.

If you agree to participate in this study, no changes in your ongoing treatment with other professionals (doctor, psychologist, occupational therapist, etc.) will be required, which must be maintained during this study. Throughout the research, we will be available to give you feedback on the results of the evaluations and to clarify any doubts you may have regarding the implication of these in your treatment.

If you accept to participate in this research, you will be invited to attend ten consecutive days for transcranial direct current stimulation and return at one month and three months after the end of the intervention for neuropsychological and psychiatric reassessments.

There is no report of serious risks associated with treatment with transcranial direct current stimulation in the literature. Possible adverse events related to the method usually include mild and transient conditions, which do not require specific intervention, such as local redness and discomfort, mild headache and, in rare cases, minor burns at the electrode placement site. However, all these events can be easily avoided by monitoring during the procedure. If you experience any discomfort or burning at the electrode application site, please inform the investigator so that he or she can turn off the power immediately.

Currently, there are no approved treatments for cognitive symptoms, such as attention / concentration difficulties, memory / learning, and problem solving, in the early stages of psychosis. Cognitive deficits, however, are strongly associated with impairments in functioning, daily activities and work. The technique of transcranial direct current stimulation has emerged as an innovative, non-invasive method, associated with minimal side effects that has presented promising preliminary results for the treatment of those symptoms.

You will not receive benefits directly because your contribution is part of an experimental study that aims to investigate the efficacy of transcranial direct current stimulation in the treatment of cognitive symptomatology in early stages of psychosis.

**1/2**

Your medical records and the information you provide will always be treated confidentially. On the other hand, always preserving your identity, the results of this study may be published in scientific journals, or taken to discussion with other health professionals. The information obtained will be analyzed together with those of other volunteers, and no research subjects will be identified.

You will also have the right to be kept up-to-date on the partial results of the surveys that are known to the researchers.

This term is being made available in 2 original ways, one to stay in your care and the other to stay with the researcher. Note that all pages are numbered, and must be initialed by the researcher and by you.

As a voluntary participant, you may drop out from the study if you wish, as well as request or access your data at any time, without any interference in the treatment you are having, or that you may need.

The researcher responsible is the Psychologist Thais Rabanea de Souza, under the guidance of Professor Acioly Luiz Tavares de Lacerda, who can be found at Rua Pedro de Toledo, 669 - 3rd floor, Vila Clementino, São Paulo / SP, telephone 11) 5576-4845, and you can contact at any time. This research was submitted to the Research Ethics Committee via ‘*Plataforma Brasil’*, Brazilian Ethics Commission, Ministry of Health. If you have any questions or concerns regarding research ethics, please contact the Research Ethics Committee (CEP) of Federal University of Sao Paulo, at Rua Francisco de Castro nº 55, Vila Clementino, CEP 04020-050, São Paulo / SP, telephone (11) 5571-1062, fax: (11) 5539-7162, e-mail: cep@unifesp.edu.br.

I declare that I am aware of the above information and that I agree to participate in this research.

Study Number:

Name of participant:

Signature of the Researcher:

Signature of participant:

Date:

**2/2**
